# De novo transcriptome assembly and characterization of nine tissues of *Lonicera japonica* to identify potential candidate genes involved in chlorogenic acid, luteolosides, and secoiridoid biosynthesis pathways

**DOI:** 10.1007/s11418-016-1041-x

**Published:** 2016-09-14

**Authors:** Amit Rai, Hidetaka Kamochi, Hideyuki Suzuki, Michimi Nakamura, Hiroki Takahashi, Tomoki Hatada, Kazuki Saito, Mami Yamazaki

**Affiliations:** 1 Department of Molecular Biology and Biotechnology, Graduate School of Pharmaceutical Sciences, Chiba University, Inohana 1-8-1, Chuo-ku, Chiba, 260-8675 Japan; 2 Kazusa DNA Research Institute, Chiba, Japan; 3 Medical Mycology Research Center, Chiba University, Chiba, Japan

**Keywords:** De novo transcriptome assembly, Chlorogenic acid, Luteolosides, Secoiridoid

## Abstract

*Lonicera japonica* is one of the most important medicinal plants with applications in traditional Chinese and Japanese medicine for thousands of years. Extensive studies on the constituents of *L. japonica* extracts have revealed an accumulation of pharmaceutically active metabolite classes, such as chlorogenic acid, luteolin and other flavonoids, and secoiridoids, which impart characteristic medicinal properties. Despite being a rich source of pharmaceutically active metabolites, little is known about the biosynthetic enzymes involved, and their expression profile across different tissues of *L. japonica*. In this study, we performed de novo transcriptome assembly for *L. japonica*, representing transcripts from nine different tissues. A total of 22 Gbps clean RNA-seq reads from nine tissues of *L. japonica* were used, resulting in 243,185 unigenes, with 99,938 unigenes annotated based on a homology search using blastx against the NCBI-nr protein database. Unsupervised principal component analysis and correlation studies using transcript expression data from all nine tissues of *L. japonica* showed relationships between tissues, explaining their association at different developmental stages. Homologs for all genes associated with chlorogenic acid, luteolin, and secoiridoid biosynthesis pathways were identified in the *L. japonica* transcriptome assembly. Expression of unigenes associated with chlorogenic acid was enriched in stems and leaf-2, unigenes from luteolin were enriched in stems and flowers, while unigenes from secoiridoid metabolic pathways were enriched in leaf-1 and shoot apex. Our results showed that different tissues of *L. japonica* are enriched with sets of unigenes associated with specific pharmaceutically important metabolic pathways and, therefore, possess unique medicinal properties. The present study will serve as a resource for future attempts for functional characterization of enzyme coding genes within key metabolic processes.

**Electronic supplementary material:**

The online version of this article (doi:10.1007/s11418-016-1041-x) contains supplementary material, which is available to authorized users.

## Introduction

*Lonicera japonica Thunb*, also known as Japanese honeysuckle, ‘Jin Yin Hua’, and ‘*Ren Dong*’, belongs to the Caprifoliaceae family and is often used in traditional Chinese and Japanese medicine [1]. *L. japonica* is native to eastern Asia, and is cultivated worldwide, particularly in China, Japan, and Korea due to its medicinal properties, and as an ornamental plant due to its pleasant smelling flowers, and attractive evergreen foliage [2]. However, as it is highly invasive to the ecology of some countries, such as New Zealand and several other countries including North America, it is considered a major nuisance and is restricted [3]. *L. japonica* has been used as traditional medicine in China for over thousands of years, and has been listed as top grade in ‘Ming Yi Bie Lu’ and ‘Shen Nong Ben Cao Jing’, and described in ‘Ben Cao Gang Mu’, the famous classical book of Chinese Materia Medica, as early as the seventeenth century, for applications in various diseases such as to clean away the heat-evil or heal the swelling [1]. Different parts of *L. japonica* have been reported to possess unique medicinal properties, with flowers and floral buds being highly used in Chinese traditional medicine, while the leaves and stems are used in Japan [2, 4, 5]. The commercial value of *L. japonica* in the herbal medicine trading market has increased by several hundred-fold in recent years, and >30 % of current traditional Chinese medicine prescriptions contain extracts from different plant parts of *L. japonica* [6]. Since 1995, *L. japonica* has been included in the Chinese Pharmacopoeia, with >500 prescriptions containing *L. japonica* being used for the treatment of various diseases [1].

Whole plant or aerial parts of *L. japonica*, particularly leaves and floral buds are used to derive bioactive metabolites for various preparations and medicinal uses [7]. Modern pharmacological studies have shown that extracts from *L. japonica* possess a wide range of bioactive properties, such as anti-bacterial, anti-inflammatory, anti-viral, anti-pyretic, anti-oxidant, anti-hyperlipidemic, and anti-nociceptive among others [2, 5, 8–15]. Extracts from *L. japonica* were used to prevent and treat severe acute respiratory syndromes, H1N1 influenza, and hand, foot and mouth diseases, and were reported to be effective against SARS coronavirus [2]. Apart from its application in traditional medicine, *L. japonica* has also been used as a health beverage such as ‘Jin Yin Hua’ tea or ‘Jin Yin Hua’ wine, as cosmetics such as ‘Jin Yin Hua’ floral water, or even as an active ingredient of toothpaste to prevent oral cavity diseases [1].

The major chemical constituents of *L. japonica* extracts include phenolic acids [16, 17], flavonoids [18, 19], volatile oils [20, 21], and saponins [22–27], and predominantly account for a wide range of attributed pharmacological properties. Chlorogenic acid (CGA) is a potent phenolic acid derived from phenylalanine and is considered to have several important biological activities. CGA, a group of esters created from certain trans-cinnamic acids such as caffeic acid, ferulic acid, and quinic acid, is a primary phenylpropanoid generated from the shikimic acid pathways with high anti-oxidant activities and, therefore, are often used in the form of medicines or foods. Studies have shown strong anti-bacterial, anti-oxidant and anti-diabetic activities attributed to CGA [1, 28, 29]. Luteolin, and its sugar-conjugated derivative, luteolosides, are also derived from phenylpropanoid metabolic pathways, and are major constituents of *L. japonica* extracts. Studies have shown luteolin and luteolosides to possess anti-oxidative, anti-inflammatory, anti-tumor, and anti-5-lipoxygenase activity [30]. CGA and luteolosides are the major constituents of *L. japonica* and are used as standard compounds for assessing its quality [28]. Besides CGA and luteolosides, secoiridoids such as loganin, secologanin, sweroside, and kingiside among others have been identified from extracts from *L. japonica*. In the past decades, >30 iridoids from *L. japonica* have been identified and reported [1, 27, 31, 32]. Iridoids and secoiridoids are pharmaceutically active metabolites, and are known to possess anti-tumor, anti-inflammatory, and anti-oxidant activities and hepatoprotective effects [32–37]. In Japanese pharmacopoeia, loganin along with CGA are recommended as a means to evaluate the quality of *L. japonica*. Several studies on chemical constituents across different tissues have shown a higher content of CGA and luteolosides in floral buds, leaves and stems of *L. japonica* [1, 5, 7]. The CGA content in *L. macranthoides*, a species closely related to *L. japonica*, was reported to be higher in young leaves and young stems compared to flowers [38]. The content of CGA, luteolosides, and other bioactive constituents of *L. japonica* varies based on tissue, extraction period or season, and their habitat.

Recent advances in next-generation sequencing, and computational resources to perform de novo transcriptome assembly and analysis has revolutionized the field of phytochemistry, especially for non-model plants [39–41]. RNA-seq-based transcriptome profiling provides a broad overview of different active metabolic processes, and their localization. Using a different statistical approach leads to the identification of potential genes involved in the pathway of interest. Previous transcriptome-based studies on *L. japonica* described transcripts across leaves and different floral developmental stages, and were focused on CGA, luteolosides, and flavonoid biosynthesis [2, 6]. However, the number of tissues used to perform de novo transcriptome assembly was limited and, therefore, does not represent a complete transcriptome for *L. japonica*. Furthermore, genes involved in secoiridoid metabolic pathways, one of the major chemical constituents with important pharmaceutical properties, have not been studied in *L. japonica*. Our study attempts to bridge this gap. We performed deep RNA sequencing for nine different tissues of *L. japonica*, yielding over 24 Gbps reads, which upon de novo transcriptome assembly, by combining three popular assemblers, resulted in 243,185 unigenes. The transcriptome assembly thus obtained is a more complete representation of the transcripts and ongoing metabolic processes of *L. japonica*. Through multiple transcriptome assemblers and integration of their resulting assemblies to obtain final de novo transcriptome assembly of *L. japonica*, we managed to capture diverse transcripts with improved N50 values and number of contigs assembled. Homologs for all enzymes from CGA, luteolin, and secoiridoid metabolic pathways were identified. Transcriptome abundance estimation across all nine tissues of *L. japonica* showed unigenes associated to key metabolic pathways were highly expressed in the young leaf and shoot apex. We also identified cytochrome P450s and UDP-glycosyl transferases, two major enzyme families involved in secondary metabolic pathways, which will serve as a basis for future validation and characterization. This study therefore presents a comprehensive transcriptome profiling and analysis for *L. japonica*, and will be useful as a resource for future functional characterization of enzymes of interest.

## Results and discussion

### Sample preparation, and Illumina sequencing

**Fig. 1:**
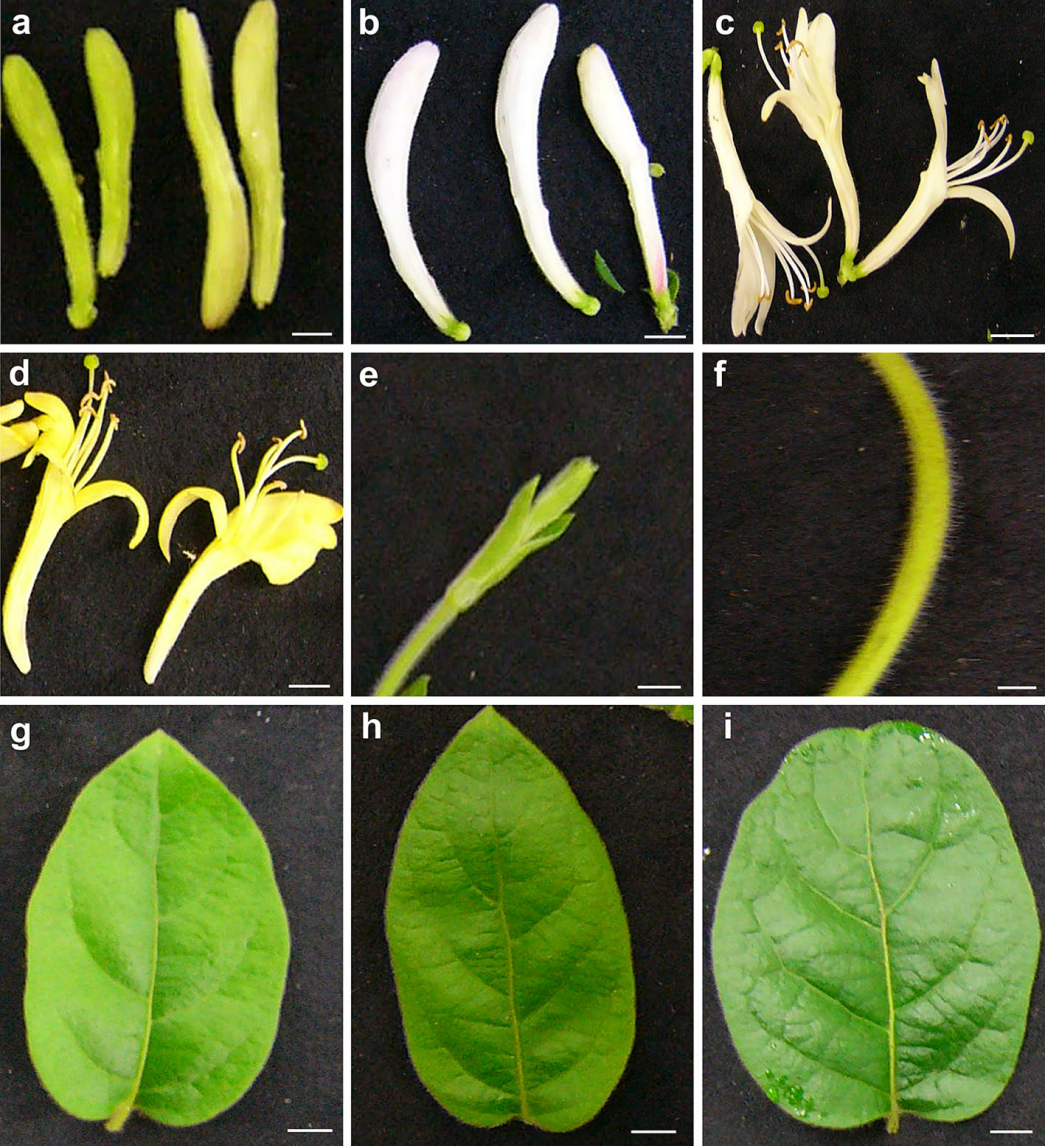
Tissues of *L. japonica* used for de novo transcriptome assembly. **a** *Green* floral buds; **b** *white* floral bud; **c** *white* flower; **d** *yellow* flower; **e** shoot apex; **f** stem; **g** leaf-1; **h** leaf-2; **i** leaf-3; the *bars* represent 1 cm

In order to achieve comprehensive representation and characterization of *L. japonica* transcriptome, we performed RNA-seq-based deep sequencing for nine tissues, namely, shoot apex, stem, leaf-1 (youngest leaf), leaf-2 (second leaf), leaf-3 (mature leaf), green floral bud, white floral bud, white flower, and yellow flower (Fig. 1). Total RNA was extracted from all nine tissues, and quality was assessed using a bioanalyzer. RNA samples with RIN (RNA integrity number) over 8 were selected for mRNA preparation, fragmentation, cDNA synthesis, and library preparation for an RNA-seq experiment. Each library, thus prepared, was sequenced using the Illumina HiSeq™ 2000 platform, yielding a total of 120 M paired-end reads with 101 bps as average sequence length.

Raw reads generated from Illumina HiSeq™ were pre-processed using the Trimmomatic program [42] for the removal of adaptor sequences, reads with a sequence length <500, low quality and ambiguous reads, yielding over 110 M paired-end clean reads, or 22 Gbps (base pairs) of reads in total (Table S1). Mean phred score, which serves as a bench mark for assessing the quality of the sequenced reads, were >36 across all nine tissues of *Lonicera japonica*; <1 % of raw reads were dropped by the Trimmomatic program based on low-quality reads or being adaptor sequences. This indicates that our RNA-seq data was of high quality, and adequate for de novo transcriptome assembly. The study overview is shown in supplementary Fig. S1.

### De novo transcriptome assembly for *L. japonica*

Success for any transcriptome-based study, especially when complete genome sequences are not available, depends upon the completeness and quality of an assembled transcriptome, which in turn depends upon the assembler program, and assembly parameters, particularly kmer size [43, 44]. Although, most de novo transcriptome assemblers rely on partitioning the sequence data into many individual de Bruijin graphs based on certain kmer size, where kmers are shorter than the reads length, output from each assembler is very different. Comparison of several popular transcriptome assemblers using different datasets revealed that none of them consistently performed to generate best assemblies [45]. Recent studies, therefore, have proposed the use of multiple assemblers in order to maximize diversity of the de novo assembled transcripts [46–48]. Therefore, in order to generate a complete de novo transcriptome assembly for *L. japonica*, we used three popular assemblers, namely, SOAPdenovo-Trans [49], Trinity [50], and CLC Genomics Workbench 8.0.3 (https://www.qiagenbioinformatics.com/).

For SOAPdenovo-Trans, we performed de novo transcriptome assemblies using different kmer values, namely, 31, 41, 51, 63, 71, and 91, resulting in six different transcriptome assemblies. Analyzing assembly stats for six de novo transcriptome assemblies generated using SOAPdenovo-Trans revealed kmer 31-based transcriptome assembly as the best, with 95,718,128 bps reads incorporated into 120,798 unigenes, with an N50 value of 1420 and the number of unigenes with sequence length >500 bps as 52,789 (43.7 %) (Table 1). The Trinity program-based de novo transcriptome assembly using default kmer (kmer as 25) resulted in the incorporation of 309,874,152 bps into 351,356 unigenes, with an N50 value of 1480 bps and the number of unigenes with sequence length >500 bps as 175,121 (49.8 %) (Table 1). For the CLC Genomics Workbench, we used default kmer size to perform de novo transcriptome assembly, resulting in assembly of 88,253,035 bps into 132,053 unigenes, with an N50 value of 975 and the number of unigenes with sequence length >500 bps as 49,831 (37.7 %) (Table 1).

**Table 1:** Summary of assembly statistics for de novo transcriptome assembly resulting from three different assemblers and their combination

	Kmer	No. of contigs	N50	Average length	Median length	Max length	*n*: >500	*n*: >1000	Total (bps)
CLC	28	132,053	975	668	393	17,676	49,831 (37.7 %)	22,776 (17.2 %)	88,253035
Trinity	25	351,356	1480	882	499	15,932	175,121 (49.8 %)	97,341 (27.7 %)	30,9874152
SOAPdenovo	31	120,798	1420	792	418	16,755	52,789 (43.7 %)	29,066 (24.1 %)	957,18128
	41	126,879	1367	761	393	19,918	52,362 (41.3 %)	28,570 (22.5 %)	965,63062
	51	131,612	1288	711	358	16,689	49,743 (37.8 %)	27,048 (20.6 %)	935,54138
	63	130,168	1222	664	324	18,719	44,775 (34.4 %)	24,670 (19.0 %)	864,87929
	71	110,659	1277	692	334	15,705	39,788 (36.0 %)	22,709 (20.5 %)	766,08845
	91	36,139	1480	946	635	12,303	20,425 (56.5 %)	12,687 (35.1 %)	341,81250
CLC_Trinity_SOAPdenovo (kmer31)_CD-HIT-EST	N.A.	243,185	1561	907	505	17,676	122,493 (50.4 %)	69,659 (28.6 %)	220,651304

The transcriptome assemblies, thus obtained from SOAPdenovo-Trans (kmer31), Trinity, and CLC Genomics Workbench were combined, and sequence redundancies were removed using the CD-HIT-EST program [51, 52], resulting in a final de novo transcriptome assembly for *L. japonica* by incorporating 220,651,304 bps into 243,185 unigenes, with an N50 value of 1561 and the number of unigenes with sequence length >500 bps as 122,493 (50.4 %) (Table 1). Comparison of transcriptome assemblies resulting from individual assemblers, or one resulting by combining the output of three assemblers showed an advantage for this approach of combining multiple assemblers, as we observed a significant gain in N50 values, average unigene length, mean unigene length, and percentage of sequences with a length >500 bps for the combined assembly. The guanine-cytosine (GC) % and length distribution for resultant transcriptome assembly for *L. japonica* is shown in Fig. S2a, b, respectively, with average GC % for all unigenes being 40.82 %, while 10,374 unigenes had a sequence length >3000 bps. The resultant de novo transcriptome assembly for *L. japonica* was improved compared to earlier published transcriptome assemblies, with a significant increase in N50 value, and overall contig length distribution.

### Functional annotation and classification of *L. japonica*

The unigene sequences derived from *L. japonica* transcriptome assembly were subjected to further characterization. A Blastx program-based [53, 54] homology search was performed for *L. japonica* unigenes against the NCBI non-redundant (nr) protein database (http://www.ncbi.nlm.nih.gov; formatted on Oct 2015) using an *E* value cut-off of <10^−5^, and the maximum number of allowed hits for each query was limited to 20. The top hit for each query sequence was used for the transcriptome annotation, and subsequent analysis and characterization. A Blastx-based homology search for *L. japonica* resulted in the annotation of 99,938 (41.1 %) unigenes (Table S2), while 143,251 unigenes had no significant sequence homology against the NCBI-nr database (Fig. S3a). Blastx results were used to extract associated gene ontology (GO) terms, to assign an enzyme commission (EC) number, and associated Kyoto Encyclopedia of Genes and Genomes (KEGG) pathway. A total of 91,745 unigenes were assigned to at least one GO term or KEGG pathway information (Fig. S3a). An *E*-value distribution plot for unigenes with a blast hit showed >89 % of aligned sequences having significant sequence homology against the NCBI-nr database (Fig. S3b). Sequence similarity distribution analysis for sequences with a blast hit showed 65,213 (65.23 %) unigenes having sequence similarity >75 % (Fig. S3c). These results, therefore, suggest that the annotation unigenes from Blastx results can be used reliably for further functional characterization.

Top-hit species distribution analysis for unigenes showed >85 % of all annotated transcripts having high sequence similarity against six plant species, namely, *Vitis vinifera*, *Populus trichocarpa*, *Ricinus communis*, *Glycine max*, *Medicago truncatula*, and *Arabidopsis thaliana* (Fig. S3d). Within these six plant species, *L. japonica* transcriptome assembly showed highest similarity to *Vitis vinifera*, with 50 % of annotated top-hit unigenes being derived from it. Top-hit species distribution results were similar to previous reports, where >50 % of annotated transcripts were assigned to *Vitis vinifera* [6].

**Fig. 2:**
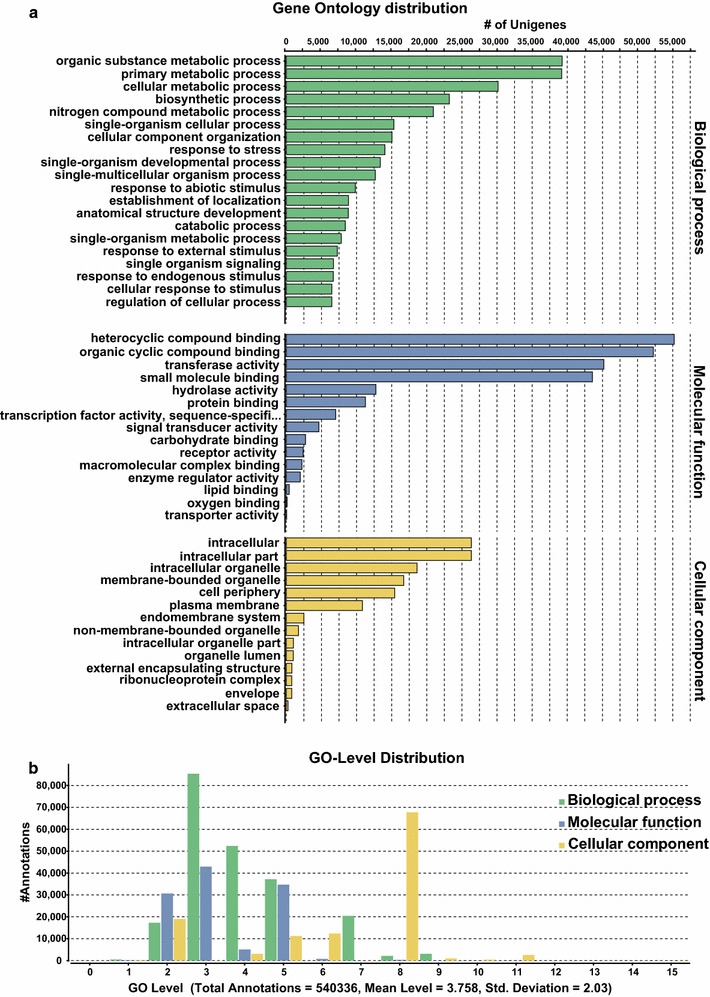
Gene ontology distribution for *L. japonica* transcriptome assembly. **a** Top 20 gene ontology terms enriched in *L. japonica* transcriptome assembly at GO-level 3 from three major categories (biological process, molecular function, and cellular component); **b** GO-level distribution for three main GO categories across *L. japonica* transcriptome assembly. GO terms for all unigenes were assigned based on Blast search results using the Blast2GO program

GO-based functional classification for *L. japonica* transcriptome assembly was performed using the Blast2GO program v 3.0 [55], resulting in a total of 178 GO categories being assigned to the annotated unigenes in three broad categories, namely, biological process (89), molecular function (53), and cellular component (36) (Table S3). GO distribution analysis by level 3, and GO level distribution for three broad categories for *L. japonica* transcriptome assembly are shown in Fig. 2a, b. Several metabolic processes, such as organic substance metabolic process, primary metabolic process, cellular metabolic process, biosynthetic process, and nitrogen compound metabolic process were the top five GO terms being enriched within *L. japonica* transcriptome. For the molecular function category, GO terms corresponding to heterocyclic compound binding, organic cyclic compound binding, transferase activity, small molecule binding, and hydrolase activity were the top five processes being enriched. For the cellular component category, GO categories corresponding to intracellular, intracellular part, intracellular organelle, membrane-bounded organelle, and cell periphery were the first five GO terms assigned to the majority of unigenes.

### KEGG database-based functional characterization of *L. japonica* transcriptome assembly

**Fig. 3:**
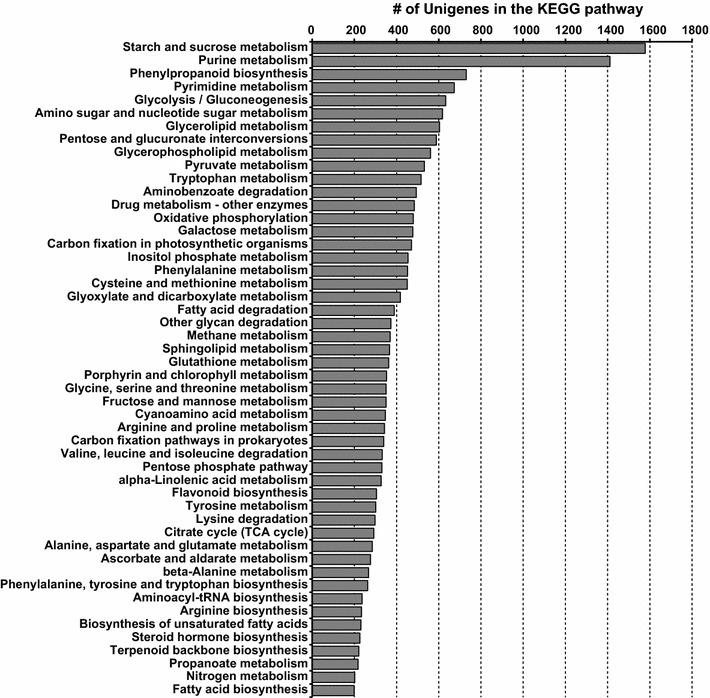
Functional annotation of *L. japonica* transcriptome assembly by KEGG pathways, with top 50 pathways based on the number of assigned unigenes

The KEGG pathway database serves as a catalog for different cellular components and their interactions within various metabolic pathways, and allows functional annotation of the unigenes by assigning their potential role in metabolic pathways [56]. Pathway-based transcriptome classification provides an overview of active metabolic processes within an organism, which when coupled with transcriptome expression analysis, gives an important insight of different metabolic processes across different tissues or conditions. Blastx results for *L. japonica* were used to assign associated KEGG pathways to all the unigenes, resulting in 25,268 unigenes grouped into 142 pathways (Table S4). The first fifty KEGG pathways based on the assigned number of unigenes are shown in Fig. 3. Starch and sucrose metabolism, purine metabolism, phenylpropanoid biosynthesis, pyrimidine metabolism, and glycolysis/gluconeogenesis were the top five KEGG pathways based on the number of assigned unigenes. Key metabolic pathways, such as phenylpropanoid metabolism, flavonoid biosynthesis, and terpenoid backbone biosynthesis, which synthesizes precursors for the biosynthesis of CGA and secoiridoids, were assigned to 231, 137, and 124 unigenes, respectively (Table S4).

### Identification of simple sequence repeats (SSRs)

SSRs are the tandem iterations of short oligonucleotides ubiquitously distributed within the genome. It consists of simple motifs of 1–6 nucleotides repeated from two to a few dozen times at a locus, and are considered as indel mutational hotspots within the genome [57, 58]. SSRs serve as an important marker for determining genetic variations, including paternity determination, population genetics studies, genetic diversity assessment, and for the development of genetic maps [57, 59]. Several reports have also implicated SSRs in affecting gene expression and, therefore, polymorphism of SSR tracts is considered important in the evolution of gene regulation [60]. In order to identify SSRs for *L. japonica*, we searched transcriptome assembly for mono- to hexa-nucleotide motifs with a minimum of ten repetitions using MISA software [61]. Overall, we identified 17,992 SSRs spread across 14,702 unigenes, with 2615 unigenes having more than one SSR (Table 2). Within the identified SSRs, mono-nucleotide represented the largest fraction (67.61 %) of all SSRs, followed by tri-nucleotides (17.95 %), and di-nucleotides (11.64 %). The number of SSRs identified as tetra-, penta-, and hexa-nucleotide repeat classes were relatively small but significant. All detected SSRs for *L. japonica* transcriptome assembly are shown in supplementary Table S5. Identified SSRs of *L. japonica* in this study may provide potential genetic markers, which will be important for population genetics and comparative genomic studies across different species or eco-types.

**Table 2:** Statistics of SSRs detected in *Lonicera japonica*

Results of SSR searches	
Total number of sequences examined	243,185
Total size of examined sequences (bp)	220,651,304
Total number of identified SSRs	98,728
Number of SSR-containing sequences	69,615
Number of sequences containing >1 SSR	20,715
Number of SSRs present in compound formation	10,315
Distribution to different repeat type classes	
Mono-nucleotides	40,746
Di-nucleotides	41,135
Tri-nucleotides	14,302
Tetra-nucleotides	1725
Penta-nucleotides	502
Hexa-nucleotides	318

### Transcriptome expression analysis for nine tissues of *L. japonica*

Transcriptome expression profiling provides a key insight into the different ongoing cellular processes under various conditions. To estimate expression abundance for unigenes across all nine tissues of *L. japonica*, clean paired-end reads were aligned to the de novo transcriptome assembly using the Bowtie 2 program [62], and transcript expression as direct count and the FPKM (fragments per kilobase of exon per million mapped fragments) values were determined by the RSEM program [63]. Among nine tissues of *L. japonica*, green and white floral bud tissues with 169,203 (69.57 %) and 160,454 (66 %) unigenes, respectively, showed the highest number of transcriptionally active unigenes (FPKM >0), while yellow flower and leaf-1 with 138,138 (56.8 %) and 141,099 (58 %), respectively, represented tissues with the lowest number of transcriptionally active unigenes (Table S6). Transcriptome expression analysis showed greater overlap in terms of transcriptionally active unigenes, with 15,551 (6.39 %) unigenes being expressed in only one of the nine tissues of *L. japonica*. Overall, FPKM value distribution for unigenes across all nine tissues was uniform except for leaf-1 and leaf-3, which showed the majority of its unigenes having lower FPKM values but the number of unigenes with an expression value >500 FPKM were highest when compared to the rest of the tissues.

**Fig. 4:**
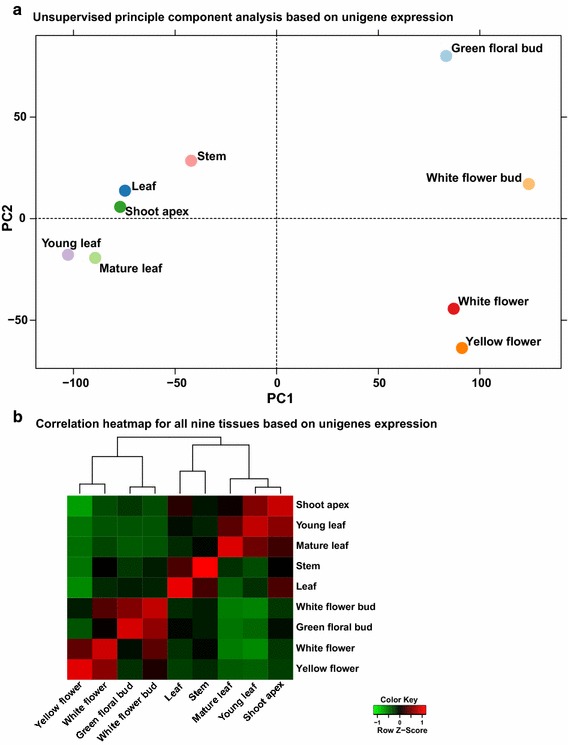
Transcriptome expression analysis across nine tissue of *L. japonica*. **a** Unsupervised principal component analysis for nine tissues of *L. japonica*; **b** correlation plot for all nine tissues of *L. japonica*. Transcriptome expression analysis for unigenes within each tissue was performed, and transcript abundance data were used to understand relationships within each tissue. Correlation between tissues based on transcript expression was calculated using Pearson’s distance matrix

In order to understand the relationship between the transcriptome dataset from all nine tissues, we used count read data to perform unsupervised principal component analysis (PCA) using the DESeq2 program [64]. The PCA plot showed all nine tissues of *L. japonica* being clustered in four major groups (Fig. 4a). Along the PC1 axis, we observed two clusters, including floral buds (green and white) and flower tissues (white and yellow) within the first group, while the second group included all leaf tissues (leaf-1,2,3), shoot apex and stem. Along the PC2 axis, these two groups were further separated to form two new groups, with flowers (white and yellow), and floral buds (green and white) being separated into two groups, while stem, shoot apex, and leaf-2 formed a separate group from leaf-1 and leaf-3. Correlation analysis based on Pearson’s distance matrix using the entire transcriptome dataset for all nine tissues was formed, and the results are shown in Fig. 4b. Similar to the PCA plot, we observed two major groups, one that included flower-related tissues while the other included all leaves and stem. Within each of these groups, we observed a higher correlation between developmentally related tissues. For example, transcripts from yellow and white flower tissues, and those from green and white floral buds were highly correlated. Green floral buds showed a high correlation with white floral buds, but a low correlation with white and yellow flowers. On the other hand, white floral buds shared a high correlation with green floral buds, and white and yellow flowers. Green floral buds represent the early development stage for flowers, which then turn into white floral buds, followed by conversion to white flowers and yellow flowers. Our PCA and correlation analysis for all nine tissues of *L. japonica* suggests the presence of signature unigenes associated with and specific to each tissue, and overlap of transcript expression across tissues was related to their association at different stages of development.

### Identification of potential candidate unigenes involved in CGA biosynthesis pathways

**Fig. 5:**
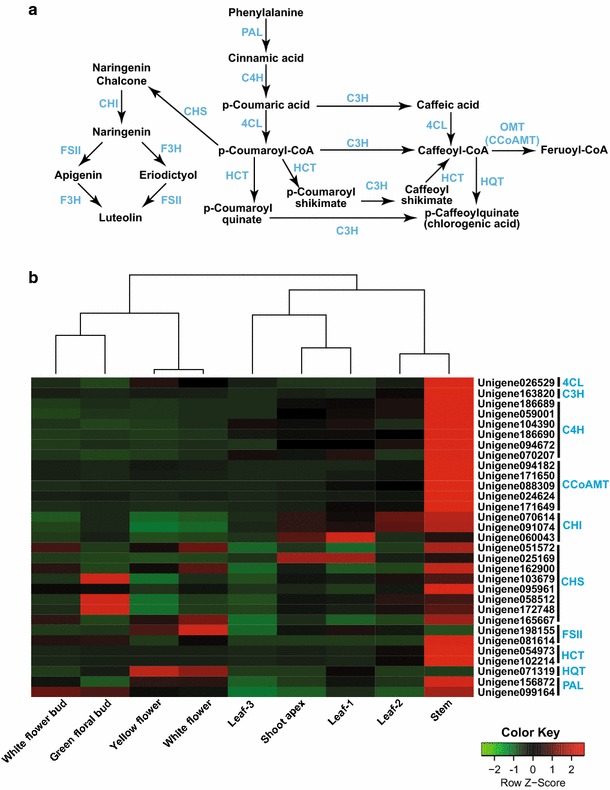
Identification of potential unigenes associated with CGA, and luteolin biosynthetic pathways, and their expression levels in different tissues of *L. japonica*. **a** Proposed CGA, and luteolin biosynthetic pathways in *L. japonica*; **b** transcript expression analysis for unigenes associated with CGA, and luteolin biosynthetic pathways. By applying a stringent filter, we were able to identify all homology in *L. japonica* transcriptome assembly for genes involved in CGA biosynthetic pathways

CGA and luteolosides, both derived from phenylalanine, are key metabolites from *L. japonica* that contribute to its medicinal properties. CGA biosynthesis is proposed to occur through three alternative routes as shown in Fig. 5a. *p*-Coumaroyl-CoA serves as an important branch point for CGA and luteoloside biosynthesis, which leads to its biosynthesis via being converted into either *p*-coumaroyl quinate, *p*-coumaroyl shikimate, or caffeoyl-CoA, while it may also lead to luteoloside biosynthesis via naringenin chalcone (Fig. 5a). To identify potential candidate unigenes from CGA and luteoloside biosynthetic pathways, we screened the annotated transcriptome assembly of *L. japonica*, and identified homologs for all known enzymes involved in these pathways. A total of 61 unigenes were identified as being associated with CGA biosynthesis pathways, while 27 unigenes were identified as being associated with luteoloside biosynthesis (Table S7). In order to narrow down the most potential candidate unigenes associated with these key metabolic pathways, we selected unigenes with lengths >500 bps and sequence similarity >90 % with a positive alignment of at least 500 bps to its corresponding blast hit. This approach resulted in the identification of 31 unigenes associated with CGA and luteoloside biosynthesis pathways (Table S8). Expression values for unigenes thus obtained were used to perform correlation analysis using Pearson’s distance matrix across all nine tissues of *L. japonica*. A correlation plot (supplementary Fig. S4) showed the formation of two clusters, with all unigenes associated with CGA except unigene 071319 (HQT) being grouped into cluster 1, and were highly correlated, while unigenes associated with luteolosides were grouped into cluster 2. Expression levels of unigenes associated with CGA biosynthesis were highly upregulated in the stem, followed by leaf-2, shoot apex, and leaf-1, while transcripts associated with luteoloside biosynthesis were highly expressed in the stem, followed by yellow flowers, white flowers, and green floral buds (Fig. 5b). The stem emerged as the tissue with the highest expression of unigenes associated with both CGA and luteoloside biosynthesis. CGA biosynthesis-associated unigenes were highly expressed in the leaf, while unigenes associated with luteolosides were highly expressed in the flowers, suggesting tissue-based preferential expression of unigenes across *L. japonica*. Previous reports on CGA accumulation in *L. macranthoides*, a closely related medicinal plant to *L. japonica*, showed a high accumulation of CGA in the young leaf, followed by the young stem, while low levels were reported in mature flowers [38].

### Identification of potential candidate unigenes involved in secoiridoid biosynthesis pathways

**Fig. 6:**
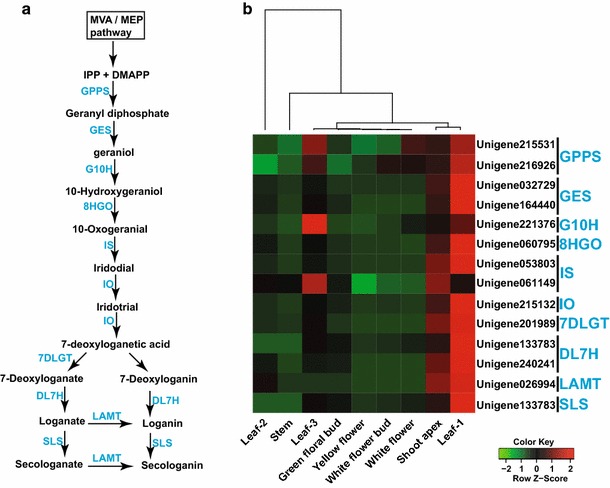
Identification of potential unigenes associated with secoiridoid metabolic pathways, and their expression levels in different tissues of *L. japonica*. **a** Proposed secoiridoid metabolic pathways in *L. japonica*; **b** transcript expression analysis for unigenes associated with secoiridoid biosynthetic pathways. Homologs for all genes from secoiridoid metabolic pathways were identified in *L. japonica* transcriptome assembly, and were highly correlated. Highest expression was observed in leaf-1 and shoot apex

Metabolites derived from secoiridoid metabolic pathways (Fig. 6a) constitute an important part of the metabolite pool of *L. japonica*, and contribute to its overall medicinal properties. Several studies have suggested anti-tumor, anti-inflammatory, and anti-oxidant activities, and hepatoprotective effects of the metabolites derived from secoiridoid pathways [1, 33–35, 37]. In order to identify unigenes associated with secoiridoid metabolic pathways, we screened annotated *L. japonica* transcriptome assembly for unigenes with sequence lengths >500 bps, and 85 % or more sequence similarity with at least 500 bps positive alignment to its closest homolog. This resulted in the identification of 24 unigenes representing all known enzymes from secoiridoid metabolic pathways (Table S9) [65]. Correlation analysis for these unigenes based on their expression values across all nine tissues of *L. japonica* using Pearson’s distance matrix showed formation of two highly correlated clusters of unigenes (Fig. S5). Among these, cluster 1, with 14 unigenes, included homologs for all enzymes associated with secoiridoid biosynthesis pathways. All these 14 unigenes were highly correlated and, therefore, were considered as potential candidate unigenes involved in secoiridoid metabolic pathways. Transcript expression analysis for unigenes associated with secoiridoid biosynthesis pathways across all nine tissues of *L. japonica* showed highest expression in leaf-1, followed by shoot apex and leaf-3 (Fig. 6b). Lower expression of unigenes associated with secoiridoid biosynthesis pathways was observed for the stem, leaf-2, floral buds (green and white), and flowers (yellow and white). While we observed high expression of CGA and luteoloside biosynthesis-associated unigenes in the stem, the same was not true for secoiridoid biosynthesis pathways. Comparing all nine tissues, leaf-1 and shoot apex emerged as key tissues which showed high expression of unigenes associated with secoiridoid, CGA, and luteoloside biosynthesis.

Cytochrome P450 (CYP) represents a large superfamily that plays an important role in oxidation and hydroxylation reactions, and is involved in key secondary metabolic pathways. UDP-glucosyl transferases (GTs) represent another super family which participates in conjugation of sugar moieties to secondary metabolites, and is responsible for huge metabolic diversity in plants. Biosynthesis of CGA, luteolin, and secoiridoids involves several CYP enzymes, while GTs play an important role in bringing metabolic diversity and regulating pools of bioactive metabolites. In this study, a total of 285 and 470 unigenes, with sequence lengths >500 bps were annotated CYPs and GTs, respectively, and are listed in Supplementary Tables 10 and 11, respectively. These unigenes are regarded as important enzyme coding genes in many secondary metabolic processes, and will serve as an important resource for future functional characterization attempts.

## Materials and methods

### Plant material preparation, RNA extraction, and library preparation

All nine tissues for *L. japonica*, namely, shoot apex, stem, leaf-1 (youngest leaf near shoot apex), leaf-2 (second leaf), leaf-3 (mature leaf), green floral bud, white floral bud, white flower, and yellow flower were harvested in June 2014. *L. japonica* plants were cultivated in the natural environment of Chiba University pharmaceutical garden, Chiba (located at 35°36′17.7″N; 140°08′06.9″E). All tissues from *L. japonica* were harvested on ice, cut into small pieces, and were snap-freezed by liquid N_2_ before storing at −80 °C prior to RNA extraction.

The frozen tissues from *L. japonica* were powdered using a multi-bead shocker (Yasui Kikai, Japan), and were used for subsequent extraction of total RNA using RNeasy Plant Mini Kit (Qiagen, USA) according to the manufacturer’s instructions. RNA quality was assessed using Agilent Bioanalyzer 2100 (Agilent Technology, USA), and RNA samples with RNA integrity number (RIN) above 8 were used for cDNA library preparation.

mRNA for each sample was isolated from the total RNA by using beads with oligo (dT), and were added with fragmentation buffer to shear mRNA into short fragments, which were then used as a template for the synthesis of first-strand cDNA using random hexamer primers. cDNA library for Illumina sequencing was prepared using SureSelect Strand specific RNA library kit (Agilent Technology, USA) according to the manufacturer’s instructions.

### Illumina sequencing and pre-processing of raw reads

A cDNA library was sequenced using Illumina HiSeq™ 2000 sequencer (Illumina Inc., USA) to obtain paired-end reads with an average length of 101 bps. cDNA library preparation and sequencing were performed at Kazusa DNA Research Institute, Chiba, Japan. The raw read sequences, transcriptome assembly, and RSEM-based transcript abundance data for nine tissues of *L. japonica* discussed in this study have been deposited in the NCBI’s Gene Expression Omnibus (GEO), and are accessible through GEO Series accession number GSE81949.

Raw reads thus obtained through Illumina sequencing were pre-processed using the Trimmomatic program [42] for the removal of adaptor sequences, empty reads, reads with ambiguous ‘*N*’ base >5 %, low-quality raw reads (Phred score <20), and raw reads with an average length <50 bps. The clean reads thus obtained were in the form of paired reads, or unpaired clean reads (forward and reverse), and were all used to perform de novo transcriptome assembly.

### De novo transcriptome assembly and transcriptome expression analysis

De novo transcriptome assembly for *L. japonica* was obtained by merging three popular assemblers, namely, SOAPdenovo-Trans, Trinity v 2.0.6, and CLC Genomics workbench v8.0.3 (https://www.qiagenbioinformatics.com/) (Qiagen, USA). For SOAPdenovo-Trans, we performed six independent de novo transcriptome assemblies using kmer sizes as 31, 41, 51, 63, 71, and 91, and resultant assemblies were analyzed using perl script from assemblathon_2 to obtain N50 values and other assembly-related stats [66]. De novo transcriptome assembly using Trinity and CLC Genomics Workbench were performed using default kmer size and default parameters. Resultant transcriptome assemblies from SOAPdenovo-Trans using kmer size as 31 emerged as the best assembly on the basis of different assembly parameters, which were then pooled together with assemblies from Trinity [50] and CLC Genomics Workbench into one merged assembly, and were processed by CD-HIT-EST v 4.6 (built on Mar 5, 2015) [51, 52] with parameters used as ‘−*c* 0.95 −*n* 8’ to remove sequence redundancy. Sequences with a length <200 bps were dropped, and the resulting de novo transcriptome assembly was used for further characterization. For transcriptome expression analysis, clean paired reads for each tissue were used for alignment over *L. japonica* transcriptome assembly using the Bowtie 2.0 program [62], and the RSEM program [63] was used for abundance estimation. To calculate unigene expression, we used the FPKM method. Unsupervised principal component analysis for all nine tissues was performed by the DESeq2 program [64] using count data for unigenes obtained from the RSEM program. GC content and basic statistic values for unigenes were calculated as described previously [67].

### Functional annotation and classification of de novo transcriptome assembly

We performed a homology search based on the Blastx program using *L. japonica* transcriptome assembly as a query against the NCBI-non redundant (nr) protein database (http://www.ncbi.nlm.nih.gov; formatted on Oct, 2015) using a cut-off *E* value of <10^−5^ with a maximum number of allowed hits of 20. The top hit for each unigene was used to annotate the transcriptome. For further characterization of *L. japonica* transcriptome assembly, we used the Blast2GO v 3.0 program [55] to assign GO terms, EC number, and KEGG pathway information to the unigenes using associated Blastx results. GO level distribution, and visualization of the top 20 GO terms from three broad categories (biological process, molecular function, and cellular component) at level 3 for *L. japonica* transcriptome assembly were performed using Blast2GO.

### Simple sequence repeat (SSR) detection

The transcriptome assembly for *L. japonica* was searched to identify the composition, frequency, and distribution of SSRs using the microsatellite identification tool (MISA) (http://pgrc.ipk-gatersleben.de/misa/) [61]. The search parameters for maximum motif length group were set to recognize hexamers with each SSR length-based category to have at least ten repeats.

## Conclusion

In this study, we performed deep RNA sequencing, and de novo transcriptome assembly for nine tissues of *L. japonica* using three popular transcriptome assemblers. With a total of 22 Gbps clean reads, transcriptome assembly for *L. japonica* was established, consisting of 243,185 unigenes with an N50 value of 1561 bps. The transcriptome assembly presented here represents much wider coverage, and longer contigs than previous reports and, therefore, improves overall transcript-associated knowledge available for *L. japonica*. Correlation-based analysis between nine tissues showed association, explaining the relationships between tissues at the developmental stages, thus suggesting that our data reliably represent transcripts for all tissues of *L. japonica* included in this study. Homologs for all genes associated with CGA, luteolin, and secoiridoid metabolic pathways were identified in *L. japonica*. Transcriptome expression analysis for unigenes associated with these key metabolic pathways revealed tissue-based transcript enrichment in *L. japonica*. Unigenes associated with CGA were highly enriched in the stem and leaf-2, while unigenes associated with luteolin were highly enriched in the stem and flowers. Transcripts from secoiridoid metabolic pathways showed the highest expression in leaf-1 and shoot apex. Our results therefore indicate that transcriptome abundance for key metabolic pathways is enriched in a tissue-dependent manner and, therefore, different tissues of *L. japonica* possess unique medicinal properties. Analyzing metabolite profiling of these tissues together with our transcriptome study to characterize relationships between gene expression and accumulation of metabolites will be highly desired for effective use of *L. japonica* as an important source of medicinal compounds. We believe that this study will serve as a milestone for functional characterization of key biosynthesis enzymes in *L. japonica*.

## Electronic supplementary material

Below is the link to the electronic supplementary material.

**Fig. S1** Experimental workflow depicting experimental design, de novo transcriptome assembly strategy, and annotation pipeline (EPS 7944 kb)

**Fig. S2** Overview of *de novo* transcriptome assembly for *L. japonica*. (a) represents  GC % distribution across *L. japonica* transcriptome assembly; (b) represents unigene length distribution across *L. japonica* transcriptome assembly (EPS 2229 kb)

**Fig. S3** Annotation for *L. japonica* transcriptome assembly using a Blastx-based homology search against the NCBI-nr protein database. (a) Blast search and annotation summary for *L. japonica* transcriptome assembly; (b) *E*-value distribution plot for unigenes with blast hit, *E*-value cut-off of <10^−5^ applied for the blastx search; (c) sequence similarity distribution plot depicting number of unigenes with a certain % sequence similarity value w.r.t. its top hit; (d) species distribution plot using top hits assigned to *L. japonica* transcriptome assembly (EPS 1876 kb)

**Fig. S4** Correlation plot for unigenes annotated as enzymes associated with chlorogenic acid and luteolin biosynthetic pathways. Correlation was measured by Pearson’s distance matrix using transcriptome abundance data for all nine tissues, which showed two clear clusters representing groups of unigenes highly correlated with each other (EPS 1710 kb)

**Fig. S5** Correlation plot for unigenes annotated as enzymes associated with secoiridoid metabolic process. Correlation was measured by Pearson’s distance matrix using transcript abundance data for all nine tissues, which showed two clear clusters representing groups of unigenes highly correlated with each other. Cluster 1 included all representative enzymes from secoiridoid metabolic pathways, and were selected for further analysis (EPS 1762 kb)

Supplementary material 6 (DOCX 15 kb)

**Table S2** Top-hit annotation for all unigenes from *L. japonica* based on Blastx results (XLSX 23361 kb)

**Table S3** Summary of the number of sequences annotated as different GO terms within three major categories (biological process, molecular function, and cellular component) in *L. japonica* transcriptome assembly (XLSX 13 kb)

**Table S4** List of KEGG pathways and the number of assigned unigenes from *L. japonica* transcriptome assembly (XLSX 13 kb)

**Table S5** All identified SSRs from *L. japonica* transcriptome assembly (XLSX 32 kb)

**Table S6** Summary of FPKM value distribution across all nine tissues of *L. japonica* (CSV 0 kb)

**Table S7** FPKM values for unigenes annotated as enzymes from CGA and luteolin biosynthetic pathways (XLSX 24 kb)

**Table S8** FPKM values for unigenes annotated as enzymes from CGA and luteolin biosynthetic pathways, and showing a high correlation with each other (CSV 6 kb)

**Table S9** FPKM values for unigenes annotated as enzymes from secoiridoid metabolic pathways (CSV 4 kb)

**Table S10** List of unigenes annotated as cytochrome P450 with sequence length >500 bps from *L. japonica* transcriptome assembly, and their expression values across all nine tissues (XLSX 79 kb)

**Table S11** List of unigenes annotated as glycosyl transferase with sequence length >500 bps from *L. japonica* transcriptome assembly, and their expression values across all nine tissues (XLSX 49 kb)

## Abbreviations

GPPS: Geranyl diphosphate synthase
GES: Geraniol synthase
G10H: Geraniol 10-hydroxylase
8HGO: 8-Hydroxygeraniol oxidoreductase
IS: Iridoid synthase
IO: Iridoid oxidase
7DLGT: 7-Deoxyloganetic acid glucosyl transferase
DL7H: Deoxyloganin 7-hydroxylase
LAMT: Loganic acid *O*-methyltransferase
SLS: Secologanin synthase
STR: Strictosidine synthase
TDC: Tryptophan decarboxylase
PAL: Phenylalanine ammonia lyase
C4H: Cinnamic acid 4-hydroxylase
C3H: *p*-Coumaric acid 3-hydroxylase
4CL: 4-Hydroxycinnamoyl-CoA ligase/4-coumarate-CoA ligase
HCT: Hydroxycinnamoyl-CoA shikimate/quinate hydroxycinnamoyl transferase
HQT: Hydroxycinnamoyl-CoA quinate hydroxycinnamoyl transferase
CHS: Chalcone synthase
CHI: Chalcone isomerase
F3H: Flavonoid 3-monooxgenase
FSII: Flavonol synthase
CCoAMT: Caffeoyl-CoA-*O*-methyltransferase
GO: Gene ontology
CGA: Chlorogenic acid
