# Supplementary material for: De novo transcriptome assembly and characterization of nine tissues of Lonicera japonica to identify potential candidate genes involved in chlorogenic acid, luteolosides, and secoiridoid biosynthesis pathways
Source: J Nat Med. 2016 Sep 14;71(1):1–15. doi: 10.1007/s11418-016-1041-x (PMC5214891; doi:10.1007/s11418-016-1041-x)
Supplement: Supplementary file 6 — Supplementary material 6 (DOCX 15 kb) [file 11418_2016_1041_MOESM6_ESM.docx]

| **Supplementary Table 1- Summary of Trimmomatic output for *Lonicera japonica***   \|  \| **Yellow flower** \| **White floral** \| **Green floral bud** \| **Stem** \| **White floral bud** \| **Mature leaf** \| **Young leaf** \| **Second leaf** \| **Shoot apex** \| \| --- \| --- \| --- \| --- \| --- \| --- \| --- \| --- \| --- \| --- \| \| **Input Read Pairs** \| 12,191,721 \| 134,93,109 \| 173,43,285 \| 12,335,635 \| 14,742,568 \| 107,90,805 \| 12,269,407 \| 10,217,277 \| 12,394,827 \| \| **Both Surviving** \| 11,008,559 (90.3%) \| 12,223,109 (90.59%) \| 15,708,477 (90.57%) \| 11,119,509 (90.14%) \| 13,378,939 (90.75%) \| 9,746,135 (90.32%) \| 11,055,436 (90.11%) \| 9,242,667 (90.46%) \| 11,217,203 (90.5%) \| \| **Forward Only Surviving** \| 827,547 (6.79%) \| 872,632 (6.47%) \| 1,139,139 (6.57%) \| 852,686 (6.91%) \| 922,196 (6.26%) \| 728,796 (6.75%) \| 833,008 (6.79%) \| 677,331 (6.63%) \| 821,014 (6.62%) \| \| **Reverse Only Surviving** \| 228,702 (1.88%) \| 261,743 (1.94%) \| 321,123 (1.85%) \| 235,274 (1.91%) \| 302,571 (2.05%) \| 205,430 (1.9%) \| 252,853 (2.06%) \| 192,740 (1.89%) \| 228,934 (1.85%) \| \| **Dropped** \| 126,913 (1.04%) \| 135,625 (1.01%) \| 174,546 (1.01%) \| 128,166 (1.04%) \| 138,862 (0.94%) \| 110,444 (1.02%) \| 128,110 (1.04%) \| 104,539 (1.02%) \| 127,676 (1.03%) \| |  |  |  |  |  |  |  |  |  |
| --- | --- | --- | --- | --- | --- | --- | --- | --- | --- | --- | --- | --- | --- | --- | --- | --- | --- | --- | --- | --- | --- | --- | --- | --- | --- | --- | --- | --- | --- | --- | --- | --- | --- | --- | --- | --- | --- | --- | --- | --- | --- | --- | --- | --- | --- | --- | --- | --- | --- | --- | --- | --- | --- | --- | --- | --- | --- | --- | --- | --- | --- | --- | --- | --- | --- | --- | --- | --- | --- |
|  |  |  |  |  |  |  |  |  |  |
|  |  |  |  |  |  |  |  |  |  |
|  |  |  |  |  |  |  |  |  |  |
|  |  |  |  |  |  |  |  |  |  |
|  |  |  |  |  |  |  |  |  |  |
